# Propolis: A Complex Natural Product with a Plethora of Biological Activities That Can Be Explored for Drug Development

**DOI:** 10.1155/2015/206439

**Published:** 2015-05-27

**Authors:** Ricardo Silva-Carvalho, Fátima Baltazar, Cristina Almeida-Aguiar

**Affiliations:** ^1^Life and Health Sciences Research Institute (ICVS), School of Health Sciences, University of Minho, 4710-057 Braga, Portugal; ^2^ICVS/3B's, PT Government Associate Laboratory, Guimarães, 4710-057 Braga, Portugal; ^3^Centre for the Research and Technology of Agro-Environmental and Biological Sciences (CITAB), Biology Department, University of Minho, 4710-057 Braga, Portugal

## Abstract

The health industry has always used natural products as a rich, promising, and alternative source of drugs that are used in the health system. Propolis, a natural resinous product known for centuries, is a complex product obtained by honey bees from substances collected from parts of different plants, buds, and exudates in different geographic areas. Propolis has been attracting scientific attention since it has many biological and pharmacological properties, which are related to its chemical composition. Several *in vitro* and *in vivo* studies have been performed to characterize and understand the diverse bioactivities of propolis and its isolated compounds, as well as to evaluate and validate its potential. Yet, there is a lack of information concerning clinical effectiveness. The goal of this review is to discuss the potential of propolis for the development of new drugs by presenting published data concerning the chemical composition and the biological properties of this natural compound from different geographic origins.

## 1. Introduction

Over the years, nature is continually surprising with diversified natural compounds that can be promising sources for the discovery of new drugs important in medicine [1]. In fact, approximately half of the currently available drugs were obtained from natural compounds or related [2]. The use of natural products as an essential route to new pharmaceutical leads is continually growing and is a research field of enormous interest because the different structural range of natural compounds can provide lead compounds for therapeutic improvement based on rationalized molecular modifications [3, 4]. It is well known that scientists have curiosity in herbs and other natural plant products for research; however only in the last years the interest in modified plant products by animals, which normally have been largely ignored and wasted, has been increased [5, 6].

Propolis, a complex mixture of compounds also called bee glue, is a natural resinous product that honeybees collect from several plants and mix it with beeswax and salivary enzymes (*β*-glucosidase) [7–11]. As inferred for the meaning of the Greek word propolis—*pro*-, for or in defence, and* polis*, the city [10]—bees use propolis on their hives as protection against predators and microorganisms, to repair damage, as a thermal isolator, and to build aseptic locals to prevent microbial infection of larvae [7, 9, 10, 12]. Since ancient times, propolis has been used by humans to meet the needs of health and food preservation [5]; but only in the last years the interest in this complex natural product has increased due to its broad spectrum of biological and pharmacological properties [12–14]. Propolis is a lipophilic material that is hard and breakable when cold but soft, flexible, and very sticky when warm; it possesses an enjoyable aromatic smell and different coloration, including brown, green, and red, among others [5, 7, 12]. In terms of chemical composition, it is generally composed of 50% resin, 30% wax, 10% essential oils, 5% pollen, and 5% other substances which include minerals and organic compounds like phenolic acids (cinnamic and caffeic acid) or their esters, flavonoids (flavones, flavanones, flavonols, and dihydroflavonols chalcones), terpenes, aromatic aldehydes and alcohols, fatty acids, stilbenes, and *β*-steroids [8, 9]. Analysis of different samples revealed that propolis chemical composition is difficult to standardize because it depends on different phytogeographic characteristics like vegetation, season, and environmental conditions of the site of collection, as bees select different plants in different habitats for propolis production [6, 8, 9].

Several* in vitro* and* in vivo* studies have been describing the plethora of biological activities and chemical profiles of propolis from different geographic origins. This review highlights published data about such works focusing on the antimicrobial, anti-inflammatory, antioxidant, immunomodulatory, and antitumor activities of different propolis types, in order to unravel the potential of this natural compound for the development of new drugs.

## 2. Use of Propolis in Ancient and Current Times

Propolis is not a new discovery. Since bee's domestication, men explore its products to their own benefit and propolis, one of the most important chemical weapons of bees, is no exception, having been employed extensively since almost immemorial times [6, 13, 15–17]. It is stated that propolis use dates back to ancient times, at least to 300 BC, where it was used in folk medicine and other activities in many parts of the world [16]. It was familiar to the Egyptians, in particular by the priests who controlled medicine and chemistry and knew very well propolis antiputrefactive properties. They learned from the bees the embalming capacity of propolis as this natural product is used to perform the mummification of corpses and prevent spread of infections. The Greek and the Roman physicians also acknowledged the potential of propolis by employing it in wound treatment, as an antiseptic and cicatrizing agent, and as mouth disinfectant. The Persians described propolis as a drug capable of acting against eczemas, myalgia, and rheumatism. Populations of the new world, like Incas, also used propolis as an antipyretic agent.

Between the 17th and 20th century, this natural product became very popular in Europe. In 1969, in the former Union of Soviet Socialist Republics (USSR), the use of propolis was accepted in human and veterinary medicine, with several applications including the treatment of tuberculosis, where the regression of lung problems and recovery of appetite were observed. Also, it was believed to cure some diseases in folk Georgian medicine. During World War II (1939–1945), doctors used propolis to treat wounds [15–17], but only in 1985, in Japan, propolis was considered as very promising in pharmacology. Before that, propolis was considered a product without market value, especially because its production is low and affects honey production. Nowadays, propolis is an important product in alternative medicine in Japan, being widely imported from Brazil [18]. In the 17th century, the Italian Antonio Stradivari who is considered the most significant and the greatest crafter of string instruments, like violins, used propolis as an ingredient in the varnish of his instruments. Curiously, such as in the 17th century, propolis is currently used in rosin for stringed instruments and in the repair of accordions [16].

Propolis is one of the few natural products that maintained popularity for a long time, although it is not considered a therapeutic agent in conventional medicine. Actually, it is widely used as a component in pharmaceutical and cosmetic products such as antiacne creams, facial and body creams, ointments and lotions, and several formulations for oral hygiene [15, 16]. It is also used in some foods and beverages, or simply as food supplement or healthy drinks. This name was attributed to the drinks because it is thought that propolis improves human health and prevents diseases such as heart disease or diabetes, among others [19].

## 3. Origin and Composition of Propolis

Propolis knowledge has registered an important evolution over time, due to exhaustive studies regarding its chemical composition and biological activities. In the 60s, it was thought that, despite its complexity, propolis chemical composition was more or less constant. Nevertheless, in the following years, analysis of a large number of samples from different geographic origins revealed that chemical composition of propolis is highly variable and also difficult to standardize because it depends on factors such as the vegetation, season, and environmental conditions of the site of collection [6, 8, 9]. Marcucci [7] and Bankova et al. [10] registered more than 300 substances in propolis and recent reports showed the presence of compounds never mentioned before [12, 17, 20, 21].

**Figure 1:**
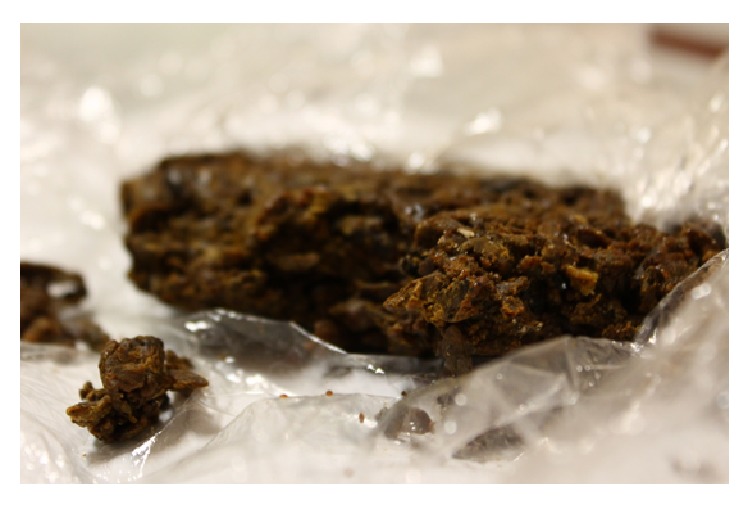
Crude sample of propolis from Pereiro, obtained in the central region of Portugal (Guarda). The sample was kindly supplied by Engineer Pedro Fernandes (Mel do Abel).

Generally, the main constituents of propolis are resin and volatiles, which are substances obtained from a variety of botanical processes in different parts of plants, and beeswax, secreted by the bees [9]. Typical compounds are summarized in Table 1. As can be seen, plant sources vary among the different parts of the globe, leading to panoply of compounds. In a review on propolis standardization, different resin types were proposed: poplar propolis, birch, green, red, “Pacific,” and “Canarian” [9, 12]. Samples of poplar propolis (e.g., from Europe, North America, New Zealand, and temperate zones of Asia) are mainly composed of flavonoids, phenolic acids, and their esters [10, 20, 22], clearly different from other propolis types (Table 1). Portuguese propolis, despite similar to the ones found in European samples, also contains new methylated, esterified, and hydroxylated derivatives of flavonoids and pinocembrin/pinobanksin derivatives containing a phenylpropanoic acid derivative moiety in their structure [20, 21]. Our group recently showed that propolis from Pereiro (district of Guarda, Beira Alta) (Figure 1) has a high concentration of phenolic components [23].

Poplar-type propolis is undoubtedly the most studied one but there are many other propolis types. Recent studies have revealed a new type of European propolis: Mediterranean propolis. This type of propolis is distinguished by the high concentration of diterpenoids and is found in many regions like Greece [24, 25], Switzerland [26], Malta [27], Turkey [28–30], or Algeria [28, 31].

Propolis from tropic regions, like Brazil, Cuba, Venezuela, and Chile, has been attracting much attention in the last years due to its particular chemical profiles. Prenylated phenylpropanoids, prenylated* p*-coumaric acids, acetophenones, diterpenic acids, and caffeoylquinic acids were shown to be very common and abundant in propolis from Brazil, mainly from the south-eastern region [9, 10, 21]. The flavonoids kaempferide and isosakuranetin and some amounts of kaempferol were also found in Brazilian samples [32]. Additionally, red propolis from the northeast regions of Brazil presents high concentrations of phenolic acids and the flavonoids formononetin, isoliquiritigenin, liquiritigenin, medicarpin, and biochanin A [33–35]. Cuban propolis has a peculiar enrichment in polyisoprenylated benzophenones, more specifically nemorosone, and a minor content of a mixture of xanthochymol and guttiferone E [9, 36] making this type of sample chemically distinct from both European and Brazilian propolis. Propolis from Venezuela is also composed of polyisoprenylated benzophenones in addition to the usual constituents found in samples of tropical regions [9, 37, 38].

Information about the chemical composition of Australian propolis is very limited despite the great biodiversity of the island. Propolis from Kangaroo Island is mainly composed of stilbenes, some of them being prenylated. Additionally, it has also some prenylated cinnamic acids and flavonoids [39], like 2′,3′,4′-trimethoxychalcone, 2′-hydroxy-3′,4′-dimethoxychalcone, 2′,4′-dihydroxy-3′-methoxychalcone, 5,7-dihydroxy-2,3-dihydroflavonol 3-acetate (pinobanksin 3-acetate), and 5,7-dihydroxy-6-methoxy-2,3-dihydroflavonol 3-acetate [40]. Western Australian propolis is composed of Xanthorrhoeol, pterostilbene, sakuranetin, and pinostrobin [41]. Propolis of Australian stingless bees (*Tetragonula carbonaria*) is composed of C-methylated flavanones [42].

“Pacific” propolis (e.g., Okinawa, Taiwan, Hawaii, Indonesia, and Myanmar) is another particular type of propolis. A new family of compounds, the prenylflavonoids, more specifically isonymphaeol-B, was identified in Okinawa propolis although three already known compounds—nymphaeol-A, nymphaeol-B, and nymphaeol-C—have also been isolated in three samples [43]. As Okinawan propolis, Hawaiian propolis is also composed of nine prenylflavonoids [44]. Taiwanese propolis is composed of eight prenylflavanones, nymphaeol-A, nymphaeol-B, and nymphaeol-C, propolins A, B, and E, isonymphaeol B, and 3′-geranyl-naringenin [45]. In another sample of the Pacific region, the Indonesian propolis, an inseparable mixture of four alk(en)ylresorcinols (5-pentadecylresorcinol, 5-(8′Z,11′Z-heptadecadienyl)-resorcinol, 5-(11′Z-heptadecenyl)-resorcinol, and 5-heptadecylresorcinol), along with four prenylflavanones, propolins D, C, F, and G, and three cycloartane-type triterpenes, mangiferolic acid, isomangiferolic acid, and 27-hydroxyisomangiferolic acid, was identified [46]. Additionally, fractioning of propolis extracts from Myanmar led to the isolation of two new cycloartane-type triterpenes, together with 13 cycloartanes and four known prenylated flavanones [47]. Thailand propolis, which might be possibly obtained from* Styrax* trees, comprises not only the typical compounds of temperate regions but also the two new phenylallylflavanones (7′′S)-8-[1-(4′-hydroxy-3′-methoxyphenyl)prop-2-en-1-yl]-(2S)-pinocembrin and (E)-cinnamyl-(E)-cinnamylidenate [48]. In propolis from Canary Islands, a different phenolic profile was found, furofuran lignans being the main compounds. Six furofuran lignans were isolated and characterized as sesamin, episesamin, methyl xanthoxylol, aschantin, sesartenin, and yangambin. Propolis also contains sugars and sugar alcohols [10, 49]. Many studies with African propolis from different regions, like Kenya, Cameroon, Congo, Oman, and Ethiopia, showed that triterpenoids are major chemical components [50–53]. Southern Nigeria propolis is uncommon since it presents prenylated isoflavonoids, like Brazilian red propolis, and a high abundance of stilbenoid compounds [54].

Propolis additionally contains minerals such as magnesium, calcium, iodine, potassium, sodium, copper, zinc, manganese, and iron; some vitamins like B1, B2, B6, C, E, and D, as well as provitamin A; a few fatty acids; and also some enzymes derived from bee glandular secretion or possibly from pollen like succinic dehydrogenase, adenosine triphosphatase, glucose-6-phosphatase, acid phosphatase, *α*-amylase, *β*-amylase, *α*-lactamase, *β*-lactamase, maltase, esterase, and transhydrogenase [17, 55]. Polysaccharides like starch and the di- and monosaccharaides glucose, fructose, ribose, rhamnose, talose, gulose, and saccharose are commonly present in propolis too [55].

## 4. Biological Properties and Therapeutic Activity of Propolis

Despite propolis popularity over time, it is not considered as a therapeutic agent in conventional medicine as the standardization of chemical composition and biological activity is lacking. Such standardization is indispensable for acceptance in the health system. Thus, characterization of different types of propolis according to its plant origin and corresponding chemical profile is mandatory. Studies about propolis bioactivity must start with chemical profiling of the extracts since that information is essential to have detailed and consistent comparative data between each type of biological activity and chemical data. This information allows extrapolating the possible activity and mechanism of action of new propolis under study and provides substantial clues for the development of new drug candidates [6, 9].

In the last decades, several studies have demonstrated the biological and pharmacological actions of different worldwide propolis samples. The following sections summarize the recent published information about antibacterial and antifungal [6, 7, 56–59], antiviral [7, 59, 60], anti-inflammatory [61], antioxidant [62–64], immunomodulatory [6, 65, 66], and antitumor activities [8, 17, 23, 64, 66, 67], revealing the interest of researchers in this bee product and its potential for the development of new drugs as well.

### 4.1. Antioxidant Activity

It is well known that an endogenous stimuli, like cellular metabolism, and exogenous agents like UV, toxins, and drugs, among others, generate reactive oxygen species (ROS), such as hydrogen peroxide (H_2_O_2_), the superoxide anion (O_2_^−^), and hydroxyl ion (HO^−^), as well as reactive nitrogen species (RNS), especially nitric oxide (NO). Carbohydrates, proteins, lipids, and nucleic acids, among other biomolecules, when exposed to the reactive species, suffer oxidative modifications that modify the cell and lead to its death [68–70]. Oxidative stress is responsible for the occurrence of a wide variety of human diseases, such as neurodegenerative [71] or cardiovascular diseases [72, 73], cancer [68, 74], diabetes [6], and atherosclerosis [75].

In the last years, several studies have been performed to evaluate the antioxidant capacity of natural products. Propolis extracts, composed of different polyphenols, have been reported to possess a potent antioxidant activity [70, 76, 77]. Additionally, the chemical varieties in different propolis samples from different regions have an influence on the antioxidant activity. Recently, Fabris et al. showed that a sample of Italian and Russian propolis ethanol extract (PEE), which have a similar polyphenolic composition, have a similar antioxidant activity, while Brazilian PEE, which have low polyphenolic composition, have a weak antioxidant activity [78]. Another study also showed, using different samples of Transylvania PEE, a positive correlation between high polyphenolic composition and high antioxidant activity [79]. Phenolic acids and flavonoids are characterized by a powerful antioxidant activity, which is closely related to the chemical structure of the compounds [80]. Briefly, the antioxidant activity is exerted by inhibiting the activity of some enzymes (e.g., xanthine oxidase, protein kinase C, ascorbic acid oxidase, cyclooxygenase, lipoxygenase, Na^+^/K^+^ ATPase, and cAMP phosphodiesterase) which inhibit the production of ROS species; by scavenging, interrupting the reactions that lead to the lipid peroxidation; by chelating metal ions, mainly iron and copper, that are involved in the process of free radical creation; or by potentiating the action of other antioxidants [55].  Table 2 summarizes some of the studies addressing the antioxidant activity of propolis.

Moreira et al. [62] and Miguel et al. [63] proposed that Portuguese propolis, an important source of total phenols, flavones, and flavonols, could be beneficial for human health due to its antioxidant properties. Portuguese propolis also protects human erythrocytes from free radicals damaging by decreasing lipid peroxidation [64]. These studies suggest that Portuguese propolis is a powerful antioxidant agent that can be used against oxidative stress, thus maintaining the structural and functional integrity of the cells. Cuesta-Rubio et al. [36] demonstrated that nemorosone, the most abundant polyisoprenylated benzophenone present in Cuban propolis, exhibits antioxidant capacity. However, when this compound suffers methylation, a process that facilitates the separation of the compound from the propolis sample, the antioxidant property is abolished. Ethyl acetate extract of Kangaroo Island propolis, which is rich in stilbenes, showed a stronger scavenging activity [39]. Yang et al. showed that ethyl acetate of propolis collected in Anhui, China, has strong scavenging activity and ferric reducing activity, those activities being influenced by caffeic acid, phenethyl caffeate, cinnamyl caffeate, and benzyl caffeate [81]. Another study showed that samples of methanolic extracts of Algerian propolis that contains high amounts of caffeic acid esters and flavanones, kaempferol, and galangin possess strong scavenging activity and ferric reducing activity [31]. Uruguay propolis with high polyphenolic composition inhibits low-density lipoprotein (LDL) peroxidation and protein nitration* in vitro*. Moreover, it induces the expression of nitric oxide synthase (eNOS) and inhibits NADPH oxidase in bovine aortic endothelial cells [82]. In another study, the topical administration of Romanian PEE in mouse, either prior to or after UVB exposure, significantly attenuated the malondialdehyde (MDA) formation and restored glutathione peroxidase (GSH-Px) activity [83]. Talas et al. [84] showed that Turkish PEE has antioxidant properties in the liver tissue of NOS inhibited rats. In fact, NOS inhibition caused an increase in CAT activity and MDA levels, effect that was significantly decreased when the rats were treated with PEE. It is well known that propolis composition is variable; nevertheless, one of its major components, CAPE (caffeic acid phenethyl ester), plays an important role in the antioxidant activity [85–87].

Antioxidant activity is one of the most studied and important activities of propolis, though there are no studies with data on the safe dose to be used in humans. Thus, clinical studies using propolis and its active compounds are needed.

### 4.2. Anti-Inflammatory Activity

Inflammation is an event that normally occurs in response to the constant exposure to environmental and endogenous stimuli as well as to accidental damage [61]. A complex cascade of chemical signals initiates after tissue injury and maintains a host response to repair the injured tissue. There are two stages of inflammation: acute and chronic. Acute inflammation is mediated through the activation of the immune system cells, which migrate to the site of damage and release growth factors, cytokines, and ROS/RNS species. Chronic inflammation occurs when the acute inflammation is not successfully resolved. This inflammatory condition plays a critical role in the pathogenesis of many diseases including atherosclerosis, cancer, asthma, Alzheimer's, and Parkinsonism [88, 89].

Several studies have associated different types of propolis and its various constituents with anti-inflammatory activity [61, 90–95].  Table 3 summarizes the anti-inflammatory mechanisms investigated with propolis and its chemical constituents. Recently, the role of the flavonoids quercetin, flavonols, and flavones in modulating inflammatory cell function was studied [92]. Funakoshi-Tago et al. [96] investigated the anti-inflammatory effects of flavonoids isolated from Nepalese PEE on the IL-33 signaling pathway. The isolated flavonoids 3′,4′-dihydroxy-4-methoxydalbergione, 4-methoxydalbergion, cearoin, and chrysin inhibited the expression of inflammatory genes including IL-6, TNF-*α*, and IL-13 in bone marrow-derived mast cells (BMMC) and also inhibited the activation of IKK, which leads to the degradation of I*κ*B*α* and inhibits the activation of nuclear factor *κ*B (NF-*κ*B).

The release and oxygenation of arachidonic acid are a critical event in inflammation. Mirzoeva and Calder [90] demonstrated that propolis components such as CAPE, caffeic acid, quercetin, and naringenin, among others, inhibit the production of eicosanoids. In fact, these components significantly suppressed the lipoxygenase pathway of arachidonic acid metabolism, CAPE being the most potent modulator. Another study indicated that CAPE treatment improves hepatic steatosis induced by high-fat diet in a mouse model. This effect was attributed to the reduction of c-Jun-N-terminal kinase (JNK1/2) and NF-*κ*B activation with decrease in COX-2 expression [97]. Recently, it was reported that CAPE exhibits inhibitory effects on the production of proinflammatory cytokines (interleukin-1*β* (IL-1*β*)), tumor necrosis factor-*α* (TNF-*α*), and monocyte chemoattractant protein 1 (MCP-1) from lipopolysaccharide- (LPS-) stimulated RAW264.7 macrophages [98]. Machado et al. [99] showed that Brazilian green propolis water extracts (PWE) modulate an anti-inflammatory cellular response in the model of LPS-induced pulmonary inflammation by decreasing the number of macrophages and neutrophils. Additionally, it induced a reduction in the secretion of IL-6 and TNF-*α* and an increase in TGF-*β* and IL-10. Another study showed that Brazilian red PEE promotes a significant decrease in renal macrophage infiltration in rats with chronic kidney disease [100]. Búfalo et al. [101] demonstrated that Brazilian PEE and caffeic acid inhibited LPS-induced NO production by RAW264.7 macrophages, acting at the transcriptional level and suggesting that their anti-inflammatory effects were mediated by downregulating NF-*κ*B, p38 MAP kinase, and JNK1/2. According to Naito et al. [95], topical application of Brazilian PEE is effective in inhibiting carrageenan-induced rat hind paw edema. This sample appears to inhibit the chemotaxis of human polymorphonuclear leukocytes (PMNs), which also contributes to its anti-inflammatory effects. Another study showed the topical anti-inflammatory activity of propolis from Chile in mice ear with induced-edema. In fact, PEE from Buin, Chile, was the most active against the inflammation induced by 12-O-tetradeca-noylphorbol-13-acetate and arachidonic acid and also inhibits significantly NO release by the macrophages [102]. More recently, Boudreau et al. [103] indicated that CAPE is a potent leukotriene biosynthesis inhibitor in PMNs that blocks 5-lipoxygenase (5-LO) activity and arachidonic acid release. A Croatian PEE may improve psoriatic-like skin lesions, which were induced in the study by irritant agents like n-hexyl salicylate or di-n-propyl disulfide, by reducing the lipid peroxidation in the skin and total number of inflammatory cells in the skin and peritoneal cavity, more specially by suppressing functional activity of macrophages [104].

### 4.3. Immunomodulatory Activity

Natural substances are considered alternative adjuvant therapies in the treatment of different diseases due to their immunomodulatory effects. Information about this type of activity was scarce for propolis until the 1990s; but published work in the last years has provided information about the influence of different propolis samples on the immune system [65, 105–112] (Table 4).

In a study using Brazilian green propolis, it was seen that the administration for 3 days of a PEE to male BALB/c mice modulated the activation of the initial steps of the immune response by upregulating toll-like receptor- (TLR-) 2 and toll-like receptor-4 expression and proinflammatory cytokines (IL-1 and IL-6) production by macrophages and spleen cells [105]. Another study demonstrated that Brazilian green PEE upregulates TLR-4 and CD80 expression in human monocytes as well as TNF-*α* and IL-10 production [113]. It was also shown that caffeic acid stimulates the activity of monocytes against* C. albicans* but it inhibits TLR-2 and HLA-DR expression as well as TNF-*α* and IL-10 production [114].

Additionally, Missima et al. [115] showed that Brazilian green PEE administered to stressed mice reduces the proinflammatory cytokines IL-1*β* and IL-6. When administered to melanoma-bearing mice submitted or not to chronic stress, it induces high levels of IL-1*β* and IL-6 and also stimulates Th1 cytokines production, indicating the activation of antitumour cell-mediated immunity. Bachiega et al. [116] investigated the immunomodulatory effect of propolis and cinnamic and coumaric acids on cytokines IL-1*β*, IL-6, and IL-10 production. Peritoneal macrophages from BALB/c mice were incubated with different concentrations of propolis (5, 50, and 100 mg/well) and coumaric and cinnamic acid (50 and 100 mg/well). Propolis and the acids stimulated IL-1*β* production and significantly inhibited IL-6 production. Then, after LPS incubation, the inhibitory concentrations of cinnamic and coumaric acids prevented efficiently its effects on IL-6 production, whereas propolis inhibited LPS effects both before and after its addition. Additionally, propolis and coumaric and cinnamic acids inhibited IL-10 production. A study which evaluated the effect of Brazilian green propolis on macrophage activation by H_2_O_2_ and NO metabolite determination [107] showed that propolis increased H_2_O_2_ generation and decreased NO generation, which favours the microbicidal activity. Recently, the immunomodulatory effect of propolis collected in Brazil was evaluated in* Leishmania (Viannia) braziliensis *experimental infection. Data shows that macrophages incubated with propolis showed a significant increase in interiorization and further killing of parasites. Also, an increased TNF-*α* production was seen in propolis-pretreated mice, whereas IL-12 was downregulated during the infection [117].

The immunomodulatory action of propolis does not occur only at the macrophage level. In fact, some studies show that this action has also an effect on lymphocyte proliferation [106, 108]. Sá-Nunes et al. [108] showed inhibitory effects of Brazilian green propolis on splenocyte proliferation, effect that was attributed to flavonoids, and enhancement effects on interferon- (IFN-) *γ* production by spleen cells. CAPE displays inhibitory effects on transcription factors NF-*κ*B and NFAT and, as a consequence, inhibits IL-2 gene transcription, IL-2 receptor expression, and proliferation of human T cells [94]. This provides new information into the molecular mechanisms involved in the anti-inflammatory and immunomodulatory activities of propolis. CAPE has various biological activities but its effect on the immunomodulatory activity remains little studied. According to Wang et al. [112], CAPE can be useful in the treatment of asthma and other allergic diseases because it can inhibit cytokines and chemokines production by human monocyte-derived dendritic cells (MoDCs), which might be related to the NF-*κ*B signalling pathway. Another study demonstrated for the first time that Brazilian propolis significantly inhibited the generation of Th1 cells. Furthermore, the effects of propolis were investigated on 2,4,6-trinitrobenzene sulfonic acid- (TNBS-) induced colitis in a mouse model. Propolis reduced the frequency of IFN-*γ*-producing CD4 T cells in a dose-dependent way under Th1-polarizing conditions. The inhibitory effect of propolis on Th1 differentiation was demonstrated* in vivo *too, and the severity of colitis in propolis-fed mice was significantly lower than that of mice fed with the control diet [118].

### 4.4. Antiviral Activity

Propolis comprises a complexity of compounds which play a role in antiviral protection. Despite the few data available regarding this activity, it was shown that propolis from different geographic regions has considerable antiviral activity by acting at different levels and interfering with the replication of some viruses [12], like herpes simplex types 1 and 2, adenovirus type 2, influenza virus, or human immunodeficiency virus (HIV), among others [7, 60, 119–124].  Table 5 summarizes the antiviral activity of propolis and its chemical constituents.

Schnitzler et al. [123] analysed the antiviral effect of PWE and PEE from Czech Republic as well as that of the constituents caffeic acid,* p*-coumaric acid, benzoic acid, galangin, pinocembrin, and chrysin, against herpes simplex virus type 1 (HSV-1) in cell culture. Both extracts exhibited high anti-HSV-1 activity when cells were treated prior to viral infection, galangin and chrysin being the main bioactive compounds. Amoros et al. [60] showed the antiviral activity of the major flavonoids of propolis, more specifically flavonols and flavones, the first being more active against HSV-1. Additionally, they analysed the effect of propolis on several DNA and RNA viruses (HSV-1, HSV-2, adenovirus type 2, vesicular stomatitis virus (VSV), and poliovirus type 2). Propolis at a concentration of 30 *μ*g/mL reduced the titer of herpes virus; however, vesicular stomatitis virus and adenovirus were less susceptible. In addition, propolis appears to exert a virucidal action on the enveloped viruses HSV and VSV [120]. Recently, it was shown that hydromethanolic extract of geopropolis from the stingless bee* Scaptotrigona postica *(Brazil) inhibits the HSV replication and also the entry of the virus into cells, effect that was attributed to the C-glycosyl flavones, catechin-3-O-gallate, and 3,4-dicaffeoylquinic acid [125]. According to Tait et al. [126], natural and synthetic flavonoids might interfere with picornavirus replication preventing the decapsidation of viral particles and RNA release within cells or blocking viral RNA synthesis. These authors also showed that different homoisoflavonoids have good antiviral activity against the coxsackie viruses B3, B4, and A9 and echovirus 30. Shvarzbeyn and Huleihel [127] tried to determine which step of Tax oncoprotein-induced NF-*κ*B activation is blocked by propolis and CAPE and showed that both substantially inhibited the activation of NF-*κ*B-dependent promoter by Tax. Also, they strongly prevented both Tax binding to I*κ*B*α* and its induced degradation by Tax. Ma et al. [128] showed that nanometer propolis flavones could significantly inhibit* in vitro* porcine parvovirus (PPV) infecting PK-15 cells and* in vivo* they restrain the PPV copy in lung, gonad, and blood, decrease the impact of PPV on weight of guinea pigs, and increase hemagglutination inhibition of PPV in serum as well as improving the contents of IL-2, IL-6, and *γ*-IFN.

Over recent years, therapeutic benefits of propolis and/or its isolated compounds have been described in HIV treatment. Ito et al. [129] tested the anti-HIV activity in H9 lymphocytes of triterpenoids melliferone, moronic acid, anwuweizonic acid, and betulonic acid and four known aromatic compounds isolated from Brazilian propolis and showed that moronic acid had significant anti-HIV activity. Gekker et al. [119] assessed the anti-HIV-1 activity of propolis in CD4^+^ lymphocytes and microglial cell cultures, observing the inhibition of viral expression in a concentration-dependent way. The possible mechanism of propolis antiviral property was suggested to involve inhibition of viral entry.

### 4.5. Antimicrobial Activity

Antimicrobial activity, one of the most studied propolis biological properties, is very well documented. This bioactivity has been largely investigated in the last years due to the need of new treatments against infectious diseases, especially with the increase of resistant pathogens to current antibiotics. Tables 6 and 7 summarise the antibacterial and antifungal activities found in propolis from different geographic origin and/or its chemical constituents.

#### 4.5.1. Antibacterial Activity

Propolis effect against several bacterial strains has evaluated [7, 56–59, 130] and supported the fact that propolis is more active against Gram-positive bacteria than Gram-negative bacteria [12, 59]. Briefly, data from different studies showed that propolis inhibits bacterial motility and enzyme activity, exhibits bacteriostatic activity against different bacterial genera, can be bactericidal in high concentrations, and affects cytoplasmic membrane [130].

Mirzoeva et al. [130] investigated the effect of PEE on the physiology of* Bacillus subtilis, Escherichia coli*, and* Rhodobacter sphaeroides*, proposing that propolis and some of its components, like cinnamic acid and flavonoids, affect the ion permeability of the inner bacterial membrane causing the dissipation of the membrane potential and inhibiting bacterial motility. A recent study [131] provided valuable information for understanding the potential anti-*H. pylori* mechanism of CAPE.* H. pylori*, a major factor for gastrointestinal illnesses, contains the enzyme* H. pylori* peptide deformylase that catalyses the removal of formyl group from the N-terminus of nascent polypeptide chains. Since the action of this enzyme is essential for* H. pylori* survival, it is considered a promising therapeutic drug target. Results from absorption spectra and crystal structural characterization showed that CAPE is a competitive inhibitor of peptide deformylase, blocking the substrate entrance and preventing substrate from approaching the active site [131].

It has been suggested that the combination of propolis with other antibiotics would allow dose reduction of selected antibiotics, thus potentiating their effect. The antibacterial activity of Italian PEE in some clinically isolated Gram-positive strains, as well as the synergetic effect with some antibiotics, was assessed by Scazzocchio et al. [56]. Italian PEE drastically increased the effect of ampicillin, gentamycin, and streptomycin and moderated the action of chloramphenicol, ceftriaxone, and vancomycin. No effect was observed when used simultaneously with erythromycin. Wojtyczka et al. [132] evaluated the* in vitro* antimicrobial activity of a Polish PEE against methicillin-sensitive* S. aureus* (MSSA) and methicillin-resistant* S. aureus* (MRSA) clinical isolates and also the combined effect of propolis with ten selected antistaphylococcal drugs. PEE displayed varying effectiveness against twelve* S. aureus* strains and potentiated the antimicrobial effect of eight antistaphylococcal against all tested strains. No synergism was observed in the case of ciprofloxacin and chloramphenicol. In another study, the effect of dichloromethane extract of French propolis against different human pathogenic bacterial strains was also tested. Although Gram-negative bacteria were not susceptible to the extract, a significant antibacterial activity against both methicillin-resistant and methicillin-susceptible* S. aureus* strains was observed [133]. The same was confirmed by Velikova et al. [28] using different PEE from Bulgaria, Greece, Turkey, and Algeria. All the samples showed a good antibacterial activity against* S. aureus* but a week or lacking effect against* E. coli*. Australian PEE from stingless bee* Tetragonula carbonaria* also inhibited the growth of* S. aureus*. Nevertheless, it was less active against* P. aeruginosa* [42]. The same was observed by Papachroni et al. [53] using PEE from Cameroon and Congo.Contrary to these studies, Katircioğlu and Mercan [134] showed that Turkish PEE was effective against Gram-negative bacteria like* E. coli.* Orsi et al. [135] investigated the possible synergism between Brazilian and Bulgarian propolis and antibiotics acting on DNA (ciprofloxacin and norfloxacin) and on metabolism (cotrimoxazole) in* Salmonella typhi*. Both samples had antibacterial activity, but no synergistic effect was detected.

Recently, the suitability of nanohydroxyapatite- (nanoHA-) based surfaces containing two Brazilian PEE (green and red ones) to prevent* S. aureus* bacterial growth and biofilm was studied. The nanoHA impregnated with the two highest concentrations (12 and 25 *μ*g/mL) of red PEE showed a remarkable reduction of 99% in the number of viable bacteria, while nanoHA with green PEE at same concentrations showed a reduction of 45 and 61%. Moreover, the nanoHA impregnated with the highest concentration of red PEE was able to inhibit 80% of the staphylococcal biofilm formation [136].

Diverse studies show that as the composition of propolis varies from region to region, the antibacterial activity also displays some variations [59]. Susceptibility of different Gram-positive bacteria to PEE varies with the place of propolis collection [137]. The antibacterial effect was shown to be higher for samples from a wet-tropical rain forest-type climate. Other studies revealed the influence of propolis geographical origin on its antibacterial properties [57, 58, 138, 139]. Propolis from the north and centre of Portugal has a great activity against* S. aureus *[58, 140].

#### 4.5.2. Antifungal Activity

Antifungal activity is also influenced by the chemical variation of propolis [59]. Several studies have shown the effect of propolis from different geographic origin against different fungi, particularly of clinical interest [141–145]. Quiroga et al. [146] demonstrated the antifungal activity of propolis from the northwest of Argentina, focusing their study on the environment and the development of agrochemicals with reduced economic costs, possibly containing propolis extracts and its isolated compounds, such as pinocembrin and galangin, as active principles.

Recently, Falcão et al. [140] screened the antifungal activity of Portuguese propolis and its potential floral sources* Populus x Canadensis* and* Cistus ladanifer* against* Candida albicans*,* Trichophyton rubrum*, and* Aspergillus fumigatus*. Plant extracts did not exhibit relevant antifungal activity, with exception of* T. rubrum,* but both propolis samples revealed similar antifungal activity, the highest being obtained against* T. rubrum* and the lowest against* A. fumigatus*. A sample of PEE from Poland showed a high fungicidal activity against* C. albicans*,* C. glabrata*, and* C. krusei *[147]. Recently, it was shown that different organic extracts of French propolis (PEE, PWE, methanolic extract, and dichloromethane extract) were effective against* C. albicans* and* C. glabrata* but only have a weak activity towards* A. fumigates* [133].

Brazilian PEE was proved to be active against several* Candida *strains (*C. albicans*,* C. tropicalis*,* C. krusei*, and* C. guilliermondii*),* C*.* albicans* being the most sensitive and* C. guilliermondii* the most resistant [141]. Brazilian green and red propolis display activity against different fungal species of* Trichophyton*, which cause dermatophytosis, red PEE being more efficient than the green one [143]. Also, it was shown that n-hexane extract of Brazilian red propolis did not induce resistance in* Candida* spp. In fact it was active against* Candida* spp. resistant to antifungal agents, like fluconazole [148]. Dota et al. [144] evaluated the* in vitro* antifungal activity of PEE and propolis microparticles (PMs) obtained from a sample from Argentina against clinical yeast isolates of importance in the vulvovaginal candidiasis.* C. albicans *and non-*C. albicans *were inhibited by PEE and PMs, with small variation. Additionally, it was shown that Brazilian green PEE has the ability to inhibit growth and biofilm formation by* C. albicans* from vulvovaginal candidiasis [149]. Another study showed fungicide action of propolis (PEE, PWE, propolis matricial microparticles (PMM), and propolis soluble dry extract after 6–8-hour treatment against all three* C. albicans* morphotypes (yeast, pseudohyphae, and hyphae), PEE being the most potent followed by PSDE, PM, and PWE [150]. Brazilian propolis induces* C. albicans* cell death mediated via metacaspase, since the metacaspase mutant in* C. albicans* showed reduced sensitivity to propolis, and by the Ras pathway. Using* C. albicans* deletion libraries, it was possible to screen several mutants in genes involved either in the morphological transitions or in the maintenance of a specific morphotype that are more sensitive to propolis. To conclude the study, the authors also showed that propolis based gels and cream were partially able to control vulvovaginal candidiasis in a mouse model [151]. These studies [144] strongly indicate that propolis has a great potential to control vulvovaginal candidiasis, representing a promising alternative therapeutic strategy.

### 4.6. Antitumour Activity

Recognition of the hallmarks of cancer affects the search and development of new methods and therapeutic agents with a sufficiently large therapeutic window to kill tumour cells while sparing normal cells. In the last years, the natural product propolis has attracted a growing interest by a large number of researchers since it contains a variety of phytochemical compounds that may act through multiple pathways to reduce the development and other malignant characteristics of cancer cells.

Recently, several* in vitro *studies have demonstrated a cytotoxic action of propolis from different geographic origin and of some of its isolated compounds on various tumour cells.* In vivo* studies also show a potential in the development of new antitumor agents, since propolis administration to mammals (e.g., rats) does not lead to detectable side effects [3]. Briefly, this natural product can block specific oncogene signalling pathways, which in turn lead to a decrease in cell proliferation and growth and can also act by decreasing the cancer stem cell population, increasing apoptosis, exerting antiangiogenic effects, and modulating the tumour microenvironment [66, 152, 153].  Table 8 summarizes the antitumour activity of propolis from different geographic origin and its chemical constituents.

Some researchers showed the effect of different types of propolis and its constituents on cancer cell growth, proliferation, and apoptosis. The hexane extract of propolis from Thailand, collected by the stingless bee* Trigona laeviceps*, which has a different behavior in propolis collection compared to the honey bees, exerts antiproliferative activity against five tested cancer cell lines (Chago, KATO-III, SW620, BT474, and Hep-G2) but not against the normal cell lines tested (HS27 fibroblast and CH-liver) [5]. A sample of PEE from Poland inhibited human malignant melanoma (Me45) and colorectal cancer (HCT 116) cells growth, as well as reduced cell size [154]. Other studies reported the antitumor activity of Brazilian PEE [155–158] which regulate the protein expression of cyclin D1, B1 and cyclin dependent kinase (CDK) as well as p21 in human prostate cancer cells, significantly affecting proliferation [158].

Although propolis containing CAPE is different from those with artepillin C, it is possible to obtain a similar inhibitory effect from both types. The effect of CAPE on different cancer cell lines was analysed and many of its effects have been shown to be mediated through inhibition of NF-*κ*B [66, 159]. CAPE can inhibit the proliferation of the colorectal cell line SW480 by decreasing the *β*-catenin, c-myc, and cyclin D1 protein expression [160]. Chuu et al. [161] observed that CAPE suppressed the proliferation of LNCaP, DU-145, and PC-3 human prostate cancer cells in a dose-dependent manner and also inhibited the tumour growth of LNCaP xenografts in nude mice. It was suggested that it acted through inhibition of p70S6K (an intermediary of the PI3K/AKT pathway responsible for the protein synthesis) and some Akt signalling networks. Wu et al. [162] demonstrated that CAPE inhibits* in vitro* and* in vivo* MCF-7 and MDA-MB-231 tumour growth without much effect on normal mammary cells by reducing the expression of growth and transcription factors, including NF-*κ*B. Recently, it was demonstrated that CAPE effect on genes that are associated with tumour cell growth and survival is related in part to its role as a histone deacetylase inhibitor [163].

As previously said, propolis can also act by decreasing the cancer stem cell population. Using the putative CD44 (+)/CD24 (−/low) breast cancer stem cells able to generate mammospheres from single cells, Omene et al. [164] showed that CAPE caused a dose-dependent inhibition of cancer stem cells self-renewal, progenitor formation, and clonal growth.

Concerning cell death, some* in vitro* studies showed different sensitivities of tumour cells to propolis extracts. PWE from Iraq inhibits the proliferation of HL-60 cells and leads to downregulation of Bcl-2 and activation of Bax [13]. Alizadeh et al. [165] investigated the protective effects of Iranian PEE on N-methyl-N-nitro-N-nitrosoguanidine- (MNNG-) initiated gastric cancer in rats. Results showed that tumour incidence, the number of lesions, structural abnormalities, and beta-catenin of the animals group treated with PEE significantly declined compared with the control. PEE also induced the expression of proapoptotic Bax and reduced antiapoptotic Bcl-2 expression. Propolis inhibits colony formation potential and promotes necrotic changes in HCT-116 cells and decreases mitotic cells and increases p53 and Ki-67 expression in HCT-116 tumor-bearing mice [13]. Szliszka's group have performed many studies to analyze the antitumour effect of different propolis and its constituents on prostate cancer cells (LNCaP and DU145) [166–168]. Brazilian green PEE sensitized these cells to TRAIL-induced death, enhanced the expression of TRAIL-R2, and decreased the activity of NF-*κ*B in LNCaP cells [167]. Cotreatment of TRAIL with artepillin C induced the significant activation of caspase-8 and caspase-3, as well as a significant disruption of the mitochondrial membrane potential [169]. Many studies have been conducted to understand the pathways involved in the apoptotic effect of CAPE. CAPE induces cell cycle arrest and apoptosis and reduces expression of NF-*κ*B in MDA-MB-231 and MCF-7 human breast cancer cells [162]. In PC3 prostate cancer cells, CAPE induced apoptosis in a dose-dependent manner that was associated with the loss of expression of the inhibitors of apoptosis: cIAP-1, cIAP-2, and XIAP [170]. Cavaliere et al. [171] showed that CAPE treatment of lymphoblastoid cell line PL104 induced apoptosis through the mitochondrial intrinsic pathway.

It has been known that cancer microenvironment is very important for carcinogenesis and it consists of stromal, endothelial, immune, and cancer cells. Natural products, like propolis and their constituents, have been shown to interfere with this symbiosis. It was demonstrated by Lee et al. [172] that CAPE could effectively suppress the adhesion and invasion potential of human hepatocellular carcinoma cells (SK-Hep1) by inhibiting the expression of MMP-2 and MMP-9 and NF-*κ*B.

Angiogenesis has a crucial role in tumour growth due to the requirement of oxygen and nutrients to sustain rapid uncontrolled proliferation and metastization. Both tumour and stromal cells can secrete proangiogenic factors that stimulate the formation and maintenance of new vessels, such as vascular endothelial growth factor (VEGF) [173]. Brazilian PEE could significantly reduce the number of newly formed vessels and suppress the proliferation of human umbilical vein endothelial cells (HUVECs) [174], this antiangiogenic effect being mainly mediated via inducing apoptosis in tube-forming endothelial cells through the inactivation of the survival signal ERK1/2 [175]. Yun et al. [176] observed that CAPE inhibited angiogenesis using the* in vivo* assay chick embryo chorioallantoic membrane (CAM). CAPE also suppresses VEGF formation by MDA-MB-231 cells and formation of capillary-like tubes by endothelial cells [162, 177]. Extracts of propolis containing artepillin C and CAPE significantly reduced the newly formed vessels and expression of MMPs and VEGF production from various cells [178] and, in accordance with this study, Izuta et al. [179] described that CAPE promotes inhibition of VEGF expression in HUVEC cells.

The first study on the antitumor activity of Portuguese propolis was only performed in 2010. Using normal and cancerous renal cells derived from human renal cell carcinoma (RCC) patients, in addition to A-498 cell line, Valente et al. [64] showed that methanolic extract of Portuguese propolis exhibited selective toxicity against malignant cells compared to normal cells and* in vitro* RCC growth was strongly inhibited. Recently, our group demonstrated the antitumour and antiangiogenic activity of the ethanol extract of Pereiro propolis (P10.EE), collected in the district of Guarda, Portugal. P10.EE affects cell viability of different tumour cells, MDA-MB-231 (breast) and DU145 (prostate) being two of the most sensitive ones, but was less cytotoxic against nontumour cells and fibroblasts. Also, it significantly decreased MDA-MB-231 and DU145 cell proliferation and migration along time, with cell cycle changes, and increased cell death. The significant increase observed in glucose consumption and lactate production could be explained in MDA-MB-231 by an increased expression of hypoxia inducible factor-1*α*, pyruvate dehydrogenase kinase, glucose transporter 1, lactate dehydrogenase, and carbonic anhydrase. Furthermore, P10.EE induced a decrease in HBMECs total biomass and proliferation and decreased vessel sprouting in the chicken chorioallantoic membrane [23].

## 5. Conclusions

From ancient to modern times, herbs and other plant products have been widely used as medicinal agents, first in folk medicine and other activities in many parts of the world and later developed and improved on a scientific basis into drugs that are used in the health system. Propolis is one of the few natural products that has maintained its popularity over a long period of time. As reviewed here, propolis contains a broad spectrum of compounds that may be useful in the treatment of different pathological conditions. In fact there is much literature that deals with the* in vitro *and* in vivo* biological properties of propolis. This wide range of bioactivities, the continuous discovery of new compounds, the long history of propolis use, and its safety profile make propolis a potential candidate for drug discovery that may be useful in several clinical scenarios. Nevertheless, it is necessary to make an effort to standardize propolis composition since it seems that propolis biological properties and chemical composition not only are variable but also are strictly linked. In our opinion, propolis extracts may be important economically and would allow a relatively inexpensive treatment in different diseases; however, to promote its use in modern medicine, it will be necessary to identify and isolate the bioactive compounds to be tested separately or in combination with other drugs already available.

Drug discovery does not consist only of the isolation of bioactive lead compounds from the natural sources. In fact, this process continues outside the academic laboratories through preclinical studies followed by clinical trials. Thus, despite the* in vitro* and* in vivo* assays, which provide new valuable information on propolis biological properties and mechanisms of action, it will be necessary to analyse the effectiveness of propolis clinically, to complement the basic research available, and to evaluate the potential of propolis in human health promotion.

## Figures and Tables

**Table 1:** Characteristic compounds of propolis from different geographic origins and respective plant source.

Geographic origin	Plant source	Typical constituents (main components)	References
Europe, North America, New Zealand, and temperate zones of Asia	*Populus *spp., more predominantly *P. nigra *	Pinocembrin, pinobanksin, chrysin, galangin, caffeic acid, ferulic acid, cinnamic acid, and their esters	[10, 20, 22]
Brazil			
Green propolis	*Baccharis *spp*.,* predominantly *B. dracunculifolia *	Prenylated phenylpropanoids, phenolic acids, prenylated *p*-coumaric acids, acetophenones, diterpenic acids, caffeoylquinic acids, kaempferide, isosakuranetin, and kaempferol	[9, 10, 32, 33]
Red propolis	*Dalbergia ecastaphyllum *	Formononetin, isoliquiritigenin, liquiritigenin, medicarpin, and biochanin A	[32, 34, 35]
Russia	*Betula *spp*., *more specifically* B. verrucosa, B. pendula*, and* B. pubescens *	Cinnamic acids, phenylpropanoid sesquiterpenols, acacetin, apigenin, ermanin, rhamnocitrin, kaempferide, *α*-acetoxybetulenol	[10, 180]
Cuba, Venezuela	*Clusia *spp., more specifically *C. rosea *and *C. minor *	Polyisoprenylated benzophenones, more specifically nemorosone, xanthochymol, and guttiferone E	[9, 36]
Mediterranean			
Greece	Probably *Conifer *spp.	Flavonoids, diterpenic acids such as isocupressic, pimaric, and communic acids, isoagatholal, agathadiol, ferruginol, 8-elemene, and totarol	[24, 25]
Switzerland	*P. tremula *	Benzyl *p*-coumarate, benzyl ferulate, and phenolic glycerides like dicoumaroyl acetyl glycerol, diferuloyl acetyl glycerol, feruloyl coumaroyl acetyl glycerol, and caffeoyl coumaroyl acetyl glycerol	[26]
Malta	*Ferula *spp*.,* most probably* Ferula communis *	Diterpenic acids such as isocupressic, communic, pimaric, and imbricatoloic acid, together with totarol and 13-epitorulosal	[27]
Turkey	*Populus *spp*., Eucalyptus* spp., and* Castanea sativa *	Pinocembrin, pinobanksin and its acetate, prenyl esters of caffeic acid, ferulic acids, diterpenic acids like pimaric, isopimaric, abietic, dihydroabietic acids, cinnamyl cinnamate, and ethyl oleate, aromatic acid esters such as benzyl cinnamate, benzenedicarboxylic acid and flavanols such as benzopyran and chrysin	[28–30]
Algeria	*Populus *spp. *Cistus *spp.	Pinocembrin, pinobanksin and its acetate, chrysin, apigenin, pectolinarigenin, pilosin, ladanein, galangin, naringenin, tectochrysin, methoxychrysin, prenyl esters of caffeic acids, ferulic acids, diterpenic acids like hydroxyditerpenic acid, labdane, and clerodane	[28, 31]
Australia			
*Apis mellifera *	*Acacia paradoxa *	Xanthorrhoeol, pterostilbene, sakuranetin, pinostrobin, stilbenes, prenylated tetrahydroxystilbenes, prenylated cinnamic acids, flavanones, flavonols, chalcones	[39–42, 181]
Stingless bee *Tetragonula carbonaria *	*C. torelliana *trees (fruit resins)		
Africa			
Nigeria	Probably *M. schweinfurthii *	Isoflavonoids, prenylated isoflavonoids, and stilbenoids	[54]
Kenya		Triterpenes, arylnaphtalene lignans such as tetrahydrojusticidin B and 6-methoxydiphyllin, geranyl stilbenes, and geranylflavon macarangin	[50]
Cameroon Congo		Triterpenes, derivatives of amyrin and lupeol and diprenyl flavonoids	[53]
Oman	*Azadirachta indica, Acacia* spp., and* Mangifera indica *	Triterpenes, prenylated flavanones such as 7-O-methyl-8-prenylnaringenin, 3′,8-diprenylnaringenin, and 8-prenyl-5,7-dihydroxy-3′-(3-hydroxy-3-methylbutyl)-4′-methoxyflavanone, chalcones, cardanol, cardols, and anacardic acids	[51]
Ethiopia	Probably* Acacia *spp.	Triterpenoids such as *α*- and *β*-amyrins, *α*- and *β*-amyryl acetates, lupeol, and *α*- and *β*-lupeyl acetates	[52]
Thailand	*Styrax *trees	Phenylallylflavanone, (E)-cinnamyl-(E)-cinnamylidenate	[48]
The Pacific region			
Okinawa, Hawaii, and Taiwan	*Macaranga tanarius *	Prenylflavonoids, more specifically isonymphaeol-B, nymphaeol-A, nymphaeol-B, nymphaeol-C, propolins, 3′-geranyl-naringenin	[43–45]
Indonesia, Myanmar	*Mangifera indica *	Alk(en)ylresorcinols, cycloartane-type triterpenes, cycloartanes, and prenylated flavanones	[46, 47]
Canary Islands	*Unknown *	Furofuran lignans	[49, 182]

**Table 2:** Antioxidant activity of propolis and its chemical constituents.

Origin	Propolis type/plant source	Type of extract/isolated compound(s)	Species/cells	Effect	References
Portugal (Serra de Bornes and Fundão)	European propolis/*Populus nigra *	Methanolic extract	Human erythrocytes	Decrease in lipid peroxidation	[64];
				Free radical scavenging	[62]

Brazil	Propolis from the stingless bee *Melipona orbignyi/*probablypoplar tree	PEE	Human erythrocytes	Free radical scavenging; inhibition of hemolysis and lipid peroxidation	[183]

Portugal (Central Algarve)	European propolis/*Populus nigra *	PEE, PWE, and methanolic extracts		Free radicals scavenging, chelation of metal ions	[63]

Cuba	Red propolis*/C. rosea *	Methanolic extract Nemorosone		Free radicals scavenging	[36]

Slovenia	European propolis/*Populus nigra *	PEE		Strong reducing power and ability to scavenge free radicals and metal ions	[184]

Romania	European propolis/*Populus nigra *	PEE	Female Swiss mice (UVB exposure)	Decrease in malondialdehyde levels, restoration of glutathione peroxidase activity	[83]

Kangaroo Island	Australian propolis/*Acacia paradoxa *	Ethyl acetate extract; stilbenes		Free radical scavenging	[39]

China	European propolis/*Populus nigra *	Ethyl acetate extract		Free radical scavenging and ferric reducing activity	[81]

Algeria	Mediterranean propolis*/Populus *spp. and * Cistus *spp.	Methanolic extract		Free radical scavenging and ferric reducing activity	[31]

Uruguay	European and green propolis/*Populus nigra *and* B. dracunculifolia *	PEE	Bovine aortic endothelial cells	Inhibition of low-density lipoprotein peroxidation and NADPH oxidase and increase in nitric oxide synthase	[82]

Brazil	Green propolis/*B. dracunculifolia *	PEE	C57BL/6 mice (acute lung inflammation caused by cigarette smoke)	Normalization of nitrite, myeloperoxidase levels, superoxide dismutase, catalase, and glutathione peroxidase activity and reduction of glutathione/oxidized glutathione ratio and malondialdehyde levels	[185]

Turkey	Mediterranean propolis/*Populus *spp*., Eucalyptus *spp., and* Castanea sativa *	PEE	Fibroblast cells	Decrease of DNA damage induced by H_2_O_2_	[186]
			Male Wistar rats	Decrease in CAT activity and MDA levels in NOS inhibited rats	[84]
			Carps (*Cyprinus carpio*)	Decrease in malondialdehyde levels, superoxide dismutase activity and increase of catalase and glutathione peroxidase activity	[187]

Purchased from Sigma Aldrich Co.	Characteristic of European type propolis	CAPE		Free radical scavenging, inhibition of xanthine oxidase activity and lipid peroxidation	[85]
			Male Wistar albino rats	Maintenance of superoxide dismutase activity, decrease of xanthine oxidase activity and malondialdehyde and nitric oxidase levels	[86]
			Peripheral blood mononuclear cells from cyclists	Reduction of hyperthermia-induced survival inhibition, necrosis, superoxide production, glutathione depletion, and intracellular superoxide	[87]

**Table 3:** Anti-inflammatory mechanisms investigated with propolis.

Origin	Propolis type/plant source	Type of extract/isolated compound(s)	Species/cells	Effect	References
Purchased: Sigma Aldrich Co. Synthesized	Characteristic of European, Brazilian, and Mediterranean propolis	Caffeic acid, quercetin, naringenin; CAPE	Peritoneal macrophages	Suppression of lipoxygenase pathway of arachidonic acid metabolism CAPE being the most potent modulator of the arachidonic acid cascade	[90]

Croatia	European propolis/*Populus nigra *	Water-soluble derivatives	Swiss albino mice	Reduction of DNA damage in peripheral lymphocytes	[188]
		PEE	Male Swiss albino mice	Suppression of functional activity of macrophages	[104]

Purchased: Sigma Aldrich Co.			J774 macrophages, Male Wistar rats	*In vitro* and *in vivo *inhibition of cyclooxygenase 1 and cyclooxygenase 2 activity	[91, 189];
			Male Wistar albino rats	Decrease of polymorphonuclear neutrophilic leukocyte infiltration in the lungs tissues	[190];
	Characteristic of European type propolis	CAPE	Gastric epithelial cell line (AGS), *H. pylori* (strain NCTC 11638)	Inhibition of *H. pylori*-induced NF-kB and activator protein 1-DNA-binding activity; prevention of IkB*α* degradation in AGS; and suppression of TNF-*α* and interleukin 8 production	[191];
Purchased: Wako Pure Chemical Industries, Ltd.			RAW264.7 macrophages	Decrease of the production of interleukin-1*β*, monocyte chemoattractant protein 1, and the production and expression of tumor necrosis factor *α* (TNF-*α*)	[98];
			Male Swiss inbred strain mice	Decrease of cyclooxygenase 2 expression, NF-*κ*B activity, c-Jun-N-terminal kinase, inhibitor of NF-*κ*B kinase (IKK), and inhibitor of NF-*κ*B (IkB) phosphorylation	[97]

Brazil	Green propolis/*B. dracunculifolia *	PEE	RAW264.7 macrophages	Downregulation of NF-*κ*B, p38 mitogen-activated protein kinase, and c-Jun-N-terminal kinase	[101]
Purchased: Acros Organics	Characteristic of European, Brazilian, and Mediterranean propolis	Caffeic acid			

Brazil	Green propolis/*B. dracunculifolia *	PEE	Sprague-Dawley rats	Inhibition of carrageenan-induced rat hind paws edema and the chemotaxis of human polymorphonuclear leukocytes (PMNs)	[95]

Synthesized	Characteristic of European type propolis	CAPE	Polymorphonuclear leukocytes obtained from Human blood	Inhibition of 5-lipoxygenase activity and arachidonic acid release	[103]

Chile	European propolis/poplar trees	PEE	Male CF-1 mice	Inhibition of NO release by the macrophages	[102]

China	European propolis/*Populus nigra *	PEE and PWE	Male ICR mice and male Wistar rats	Inhibition of the activation and differentiation of mononuclear macrophages; decrease prostaglandin-E2 (PGE 2) and nitric oxide (NO) levels	[61]

Brazil	Green propolis/*B. dracunculifolia *	PWE	Swiss and BALB/c mice	Decrease in the number of macrophages and neutrophils; inhibition of proinflammatory cytokines and increase of anti-inflammatory cytokines	[99]
	Red propolis/*Dalbergia ecastaphyllum *	PEE	Male Wistar rats	Decrease in renal macrophage infiltration in rats with chronic kidney disease	[100]

Nepal	Nepalese propolis/probably *Shorea robusta, Dalbergia sissoo, Acacia catechu, *and* Bombax cieba *	PEE, 3′,4′-dihydroxy-4-methoxydalbergione, 4-ethoxydalbergion, cearoin, and chrysin	Bone marrow-derived mast cells (BMMC) from C57BL/6 mice	Inhibiting IL-6, TNF-*α*, and IL-13 gene expression in BMMC and also inhibiting the activation of IKK leading to NF-*κ*B inactivation	[96]

**Table 4:** Immunomodulatory activity of propolis and its chemical constituents.

Origin	Propolis type/plant source	Type of extract/isolated compound(s)	Species/cells	Effect	References
Brazil	Green propolis/*B. dracunculifolia *	PEE	Male BALB/c mice	Upregulation of toll-like receptor-2 and receptor-4 expression and increases in interleukin-1 and interleukin-6 production	[105]
				Upregulation of toll-like receptor-2 and receptor-4 mRNA expression	[111]
			Male C57BL/6 mice, B16F10 cell line	Stimulation of the expression and production of interleukin-2 and interleukin-10 and Th1 cytokine (interleukin-2 and IFN-*γ*) production	[192]
			Male BALB/c mice	Inhibition of Th1 cells generation; reduction of the frequency of IFN-*γ*-producing CD4^+^ T cells under Th1-polarizing conditions	[118]
			Male BALB/c mice	Increase of H_2_O_2 _generation and decreases in the NO generation in peritoneal macrophages	[109]
			Male BALB/c mice	Increase in the interiorization and killing of the parasites *Leishmania (Viannia) braziliensis *by macrophages; increase in TNF-*α* production and decrease in interleukin-12 production	[117]
			Monocytes from human blood	TLR-4 and CD80 expression in human monocytes as well as TNF- *α* and IL-10 production	[113]
			Melanoma cells (B16F10); male C57BL/6 mice	Reduction of IL-1*β* and IL-6 in LPS-stressed mice; induction of IL-1*β* and IL-6 and Th1 cytokines in melanoma-bearing mice submitted or not to chronic stress	[115]

Brazil Purchased: Acros Organics	Green propolis/*B. dracunculifolia* Characteristic of European, Brazilian, Russian, Mediterranean, and Australian type propolis	PEE, cinnamic and coumaric acids	Male BALB/c mice	Stimulation of interleukin-1*β* production and inhibition of interleukin-6 and interleukin-10 productions	[116]

Purchased: Acros Organics	Characteristic of European, Brazilian, and Mediterranean propolis	Caffeic acid	Monocytes from human blood	Stimulation of monocytes activity against *C. albicans*; downregulation of TLR-2 and HLA-DR expression and inhibition of cytokine production	[114]

Purchased: Acros Organics	Characteristic of European, Brazilian, Russian, Mediterranean, and Australian type propolis	Cinnamic acid	Monocytes from human blood	Downregulation of toll-like receptor-2, HLA-DR molecules from human antigen-presenting cells, and CD80; upregulation of toll-like receptor-4, inhibition of TNF-*α* and interleukin-10 production	[193]
Purchased: Sigma Aldrich Co.		Cinnamic acid	Female IRC mice	Increase of lymphocyte proliferation and release of cytokines interleukin-1 and interleukin-2	[106]

Brazil	Green propolis/*Baccharis dracunculifolia *	Hydroalcoholic (HPE) solution	Male BALB/c mice	Increase of H_2_O_2 _generation and decrease of NO generation	[107]
			Male BALB/c mice	Decrease of splenocyte proliferation and increase of IFN-*γ* production by spleen cells	[108]

Indonesia	The Pacific region propolis/*Macaranga tanarius* and *M. indica *	HPE	Male BALB/c mice	Increase of IgG generation and macrophage phagocytosis activity and capacity	[110]

Turkey	Mediterranean propolis*/Populus *spp*., Eucalyptus *spp., and* Castanea sativa *	PEE	Peripheral blood mononuclear cells from healthy humans	Suppression of neopterin release and tryptophan degradation, downregulation of the enzyme indoleamine 2,3-dioxygenase (IDO) and decrease of IFN-*γ* and TNF-*α* levels	[194]

Purchased: Sigma Aldrich Co.	Characteristic of European type propolis	CAPE	Human monocyte-derived dendritic cells (MoDCs) generated from peripheral monocytes	Inhibition of IL-12 p40, IL-12 p70, IL-10, IFN-*γ*-inducible protein- (IP-) 10 levels; inhibition of I*κ*B*α* phosphorylation and NF-*κ*B activation	[112]
			Female BALB/c mice	Increase of IgM antibody production, T lymphocyte proliferation, interleukin-4 and interleukin-2 production by splenocytes, and IFN-*γ* production	[195]
			Human peripheral blood mononuclear cells, jurkat cells	Inhibition of transcription factors NF-*κ*B and NFAT; inhibition of interleukin-2 gene transcription, interleukin-2 receptor expression, and proliferation of human T cells	[94]

**Table 5:** Antiviral activity of propolis and its chemical constituents.

Origin	Propolis type/plant source	Type of extract/isolated compound(s)	Species/cells/viruses	Effect	References
Purchased: Sigma Aldrich Co.	Characteristic of European type propolis	Caffeic acid, *p*-coumaric acid, benzoic acid, galangin, pinocembrin, and chrysin	RC-37 cells, herpes simplex virus type 1 (HSV-1) strain KOS	High anti-HSV-1 activity for both extracts when cells were treated prior to viral infection	[123]
Czech Republic	European propolis/*Populus nigra *	PEE and PWE	RC-37 cells, herpes simplex virus type 2 (HSV-2)	High antiherpetic activity for both extracts when viruses were pretreated prior to infection	[196]

Brazil	Brown propolis/*B. dracunculifolia *	HPE	HSV-2 strain propagated in Vero cells, female BALB/c mice	Effective against HSV-2 infection and in reducing extravaginal lesions by acting on inflammatory and oxidative processes; reducing reactive species, tyrosine nitration, ascorbic acid levels, and myeloperoxidase activity and protecting against inhibition of catalase activity	[124]
	Characteristic of Brazilian propolis	Isopentyl ferulate (isolated from an PEE)	Influenza viruses A/PR/8/34 (H1N1), A/Krasnodar/101/59 (H2N2), and A/Hong Kong/1/68 (H3N2)	Suppression of influenza virus A/Hong Kong reproduction *in vitro *	[121]
	Green propolis/*B. dracunculifolia, B. erioclada, Myrceugenia euosma *	PEE	Influenza A/PR/8/34 (H1N1) virus propagated Madin-Darby canine kidney (MDCK) cells, female DBA/2 Cr mice	Reduction of body weight loss of infected mice and virus yields in the bronchoalveolar lavage fluids of lungs	[122]

France	European propolis/*Populus nigra *	PEE	RC-37 cells, HSV-1 strain H29S, acyclovir resistant mutant HSV1-R strain H29R, HSV-2, adenovirus type 2, poliovirus type 2, and vesicular stomatitis virus (VSV)	Reduction of titre of herpes virus, being vesicular stomatitis virus and adenovirus less susceptible; virucidal action on the enveloped viruses HSV and VSV	[120]

Brazil	Geopropolis from the stingless bee *Scaptotrigona postica *	Hydromethanolic extract	African green monkey kidney cells (ATCC CCL-81); herpes simplex virus strain (McIntyre)	Inhibition of HSV replication and entry into cells	[125]

Synthesized	Characteristic of Brazilian red and green propolis	Homoisoflavonoids, specially 3-benzyl-4-chromones	BGM (Buffalo Green Monkey) cells, coxsackie viruses B3, B4, and A9 and echovirus 30	Good antiviral activity against the coxsackie viruses B3, B4, and A9 and echovirus 30	[126]

Canada	European propolis/*P. trichocarpa *and* P. tremuloides *	PEE	HSV-1 and HSV-2 virus replicated in MDBK (monolayer cultures of Madin-Darby bovine kidney) cells	Impairing the ability of the virus to adsorb or to penetrate the host cells	[197]

Brazil	Green propolis/*Baccharis dracunculifolia *	Water extracts	Female BALB/c mice, Influenza A virus strain A/WSN/33 (H1N1)	Extension of the lifetime of mice. 3,4-dicaffeoylquinic acid which increases mRNA levels of tumor necrosis factor-related apoptosis-inducing and decreases H1N1 hemagglutinin mRNA	[198]
	Characteristic of Brazilian green propolis	3,4-Dicaffeoylquinic acid (Isolated from Brazilian propolis)			

Brazil	Characteristic of Brazilian green propolis	Melliferone, moronic acid, anwuweizonic acid, and betulonic acid (isolated from Brazilian propolis)	H9 lymphocytes, HIV-1	Moronic acid inhibiting anti-HIV replication	[129]

Israel	Mediterranean propolis*/Populus *spp*., Eucalyptus *spp., and *Castanea sativa *	PWE	Jurkat, uninfected human T-cell lines, and MT2 (HTLV-1 infected human T cells) cells	Inhibition of the activation of NF-*κ*B-dependent promoter by Tax and prevention of Tax binding to I*κ*B*α* and its degradation	[127]
Purchased: Sigma Aldrich Co.	Characteristic of European propolis	CAPE			

Provided by Binzhou Animal Science and Veterinary Medicine Academy of Shandong Province		Nanometer propolis Flavone	Kidney cells (PK-15) Porcine parvovirus (PPV) Britain White guinea pigs	Inhibition of PPV infecting porcine kidney- (PK-) 15 cells Restraining of PPV copy in lung, gonad, and blood, decrease of the impact of PPV on weight of guinea pigs, and increase of hemagglutination inhibition of PPV in serum as well as improving the contents of IL-2, IL-6, and *γ*-IFN	[128]

USA and China	European propolis/*Populus nigra *	PEE	Peripheral blood mononuclear cells obtained from blood of healthy donors, microglial cells isolated from human fetal brain tissue, HIV-1_AT_, HIV-1_SF162_	Inhibition of HIV-1 variants expression	[119]
Brazil	Green propolis/*Baccharis dracunculifolia *				

**Table 6:** Antibacterial activity of propolis and its chemical constituents.

Origin	Propolis type/plant source	Type of extract/isolated compound(s)	Species	Effect	References
Purchased: Bee Health Ltd. (Scarborough, Yorkshire, UK);	European propolis/*Populus nigra; *	PEE	*B. subtilis *SG38, *E. coli*, and *R. sphaeroides *	Influencing the ion permeability of the inner bacterial membrane; Inhibition of bacterial motility	[130]
Purchased: Sigma Chemical Co. (Poole, Dorset, UK)	Characteristic of European, Brazilian, and Mediterranean propolis	Caffeic acid, CAPE, quercetin, and naringenin			

Greece	Mediterranean propolis/probably *Conifer *spp.	Terpenes (isolated from Cretan propolis)	*S. aureus* (ATCC 25923), *S. epidermidis *(ATCC 12228), *E. coli *(ATCC 25922), *E. cloacae* (ATCC13047), *K. pneumoniae* (ATCC 13883), and *P. aeruginosa* (ATCC 227853)	Influencing the Gram-positive and Gram-negative bacteria viability	[199]

France	European propolis/*Populus nigra *	Dichloromethane extract	Gram-negative: 7 *Acinetobacter baumannii* (RCH, SAN008, 12, AYE, CIP7034, 107292, and 5377), 2 *Escherichia coli *(ATCC 25922 and a clinical isolate), 3 *Pseudomonas aeruginosa* (ATCC 27853 and two clinical isolates), and 4 clinical isolates of *Enterobacter cloacae*, *E. aerogenes*, *Klebsiella oxytoca*, and *Salmonella enteritidis* (phage type 4) Gram-negative: 13 *Staphylococcus aureus* (ATCC 25923, six methicillin-susceptible clinical isolates, and six methicillin-resistant clinical isolates), 2 clinical isolates of *S. epidermidis* (methiS and methiR), 3 clinical isolates of *Enterococcus faecalis* and 1 *E. faecium*, and 1 clinical isolate of *Corynebacterium striatum *	Influencing the Gram-positive bacteria viability specially *S. aureus* and several of its methicillin-resistant and methicillin-susceptible	[133]

Bulgaria	Mediterranean propolis	PEE	*S. aureus 209; E. coli WF+, *	Decrease of *S. aureus* growth and weak or lack of activity against *E. coli *	[28]
Greece	*Populus *spp.				
Turkey	*Conifer *spp.				
Algeria	*Populus *spp*., Eucalyptus *spp., and *Castanea sativa Populus *spp. *Cistus *spp.				

Australia	Australian propolis from stingless bee *Tetragonula carbonaria/C. torelliana *trees (fruit resins)	PEE	*S. aureus *(ATCC 25923); *P. aeruginosa *(ATCC 27853)	Inhibition of *S. aureus *growth	[42]

Cameroon and Congo	African propolis/probably *M. schweinfurthii *	PEE	*S. aureus* (ATCC 25923), *S. epidermis *(ATCC 13047), *E. coli *(ATCC 25922), *Klebsiella pneumonia* (ATCC 13883), and *P. aeruginosa* (ATCC 227853)	Inhibition of *S. aureus *growth	[53]

Brazil	Green propolis/*B. dracunculifolia *	PEE	*S. aureus* 2979 and *S. aureus* 4118 isolated from mastitic cows, *S. aureus* (ATCC 29213)	Decrease of *S. aureus* growth in complex media and killing of *S. aureus* cells resuspended in milk; promotion of changes in morphology and cell size	[200]

Czech Republic	European propolis/*Populus nigra *	Dimethylsulfoxide extract	*S. aureus *(CAPM 5970), *E. faecalis* (CAPM 5613 (EBF/30/39)), *E. coli *(CAMP 3101^T^ (U 5/41)), and *L. monocytogenes* (CCM 5580)	Different concentrations affect the growth of the tested bacteria	[201]

Italy	Mediterranean propolis/*Crupessus *spp.	PEE	*Staphylococcus* spp. strains (35 *S. aureus*, 63 *S. epidermidis*, 7 *S. hominis*, 18 *S. haemolyticus*, 10 *S. warneri*, 4 *S. capitis*, and 3 *S. auricularis*) and *Streptococcus* spp. strains (59 *S. faecalis*, 30 *S. viridans*, 15 *S. β-haemolyticus*, and 19 *S. pneumoniae*)	Complete suppression of the factor coagulase, reduction of lipase and prevention of biofilm formation of *Staphylococcus*; increase of the effect of ampicillin, gentamycin, and streptomycin and moderating the action of chloramphenicol, ceftriaxone, and vancomycin	[56]

Brazil	Red propolis/*D. ecastophyllum;* Green propolis/*B. dracunculifolia *	PEE	*S. aureus *(ATCC 25923)	NanoHA matrix with red and green propolis which reduces bacterial growth and biofilm formation, the nanoHA with red propolis being the most efficient	[136]

Poland	European propolis/*Populus nigra* and some species of *Betula alba, Alus glutinosa, Aesculus hippocastanum, Fagus sylvatica *		Coagulase-positive *S. aureus* strains isolated from blood clinical samples, *S. aureus *(ATCC 25923), *S. aureus* (ATCC 43300), methicillin-sensitive and resistant *S. aureus *	Inhibition of *S. aureus *growth and bactericidal activity; potentiation of antistaphylococcal drugs action; effective against twelve *S. aureus *strains, with MIC values within 0.39 to 0.78 mg/mL and MBC within 0.78 to 3.13 mg/mL	[132]

Brazil; Bulgaria	Green propolis/*B. dracunculifolia*; Mediterranean propolis/*Populus *spp.;	PEE	*S. typhi* (00238)	Brazilian propolis having bacteriostatic activity; Bulgarian propolis having bactericidal activity; both having similar synergetic effect when in combination with amoxicillin, ampicillin, and cephalexin	[202]
			*S. typhimurium* Male BALB/c mice	Increase of bactericidal activity of macrophages	[203]
			*S. typhimurium *	Both samples having antibacterial activity but no synergistic effects with ciprofloxacin, norfloxacin, and cotrimoxazole	[135]

Turkey	*Mediterranean propolis/Populus *spp*., Eucalyptus* spp. and *Castanea sativa *	PWE	*M. tuberculosis* (H_37_R_v_), male guinea-pig	Inhibition of tuberculosis infection in guinea-pigs since it promotes a decrease in necrosis formation and increase in granuloma formation	[204]
		PEE	*E. coli *(ATCC 35218)*, K. pneumoniae *(ATCC 27736)*, P. aeruginosa *(ATCC 27853)*, Morganella morganii (clinical isolate), S. aureus *(ATCC 25923)*, B. subtilis *(ATCC 6633), and *Proteus vulgaris *	Inhibition of Gram-negative bacteria growth	[134]
Purchased from Sigma Aldrich Co.	Characteristic of European propolis	CAPE	*H. pylori *	Competitive inhibitor against *H. pylori* peptide deformylase, blocking substrate entrance	[131]

**Table 7:** Antifungal activity of propolis and its chemical constituents.

Origin	Propolis type/plant source	Type of extract/isolated compound(s)	Species/cells	Effect/stimulus	References
Brazil	Green propolis/*B. dracunculifolia *	PEE	*C. albicans, C. tropicalis, C. krusei, *and *C. guilliermondii*; adult volunteer patients showing symptoms of stomatitis	Inhibition of cell growth, *C. albicans *being the most sensitive and *C. guilliermondii *the most resistant; reduction of the number of *Candida* yeasts in the saliva	[141]
	Green propolis/*B. dracunculifolia; *red propolis/*D. ecastaphyllum *	PEE	*T. rubrum, T. tonsurans, T. mentagrophytes,* and* T. mentagrophytes* (ATCC 9533) (control)	Both samples which decrease cell growth, red PEE being more efficient than the green one	[143]
	Green propolis/*B. dracunculifolia *	PEE, PWE, matricial microparticles, and soluble dry extract	*C. albicans* strains SC5314 (wild type), CAI4, and 3153A (wild type), female BALB/c mice	PEE being the most potent in inhibiting cell growth followed by propolis soluble dry extract, propolis matricial microparticles, and PWE Different gel formulations of propolis: propolis based Carbopol 940 gel (CP1%), propolis based poloxamer 407 gel with Carbopol 940 (PP1%), propolis alginate with pectin (AlP1%), and propolis based chitosan gel with Natrosol (ChP1%); CP1% and chitosan gels being the most pseudoplastic ones; propolis based gels presenting antifungal action similar to clotrimazole cream	[150]
	Green propolis/*B. dracunculifolia *	PEE, gels, and cream obtained from the extract	*C. albicans* strains used were SC5314, CAI4, BWP17, DAY286, 3153A, and 529L (wild type), female BALB/c mice (murine model of vulvovaginal candidiasis)	Induction of cell death in *C. albicans *mediated via metacaspase and RAS pathway Inhibition of all three *C. albicans* morphogenetic types, several mutants in genes involved either in the morphological transitions or in the maintenance of a specific morphotype which are more sensitive to propolis Propolis based gels and cream which were partially able to control vulvovaginal candidiasis	[151]
	Green propolis/*B. dracunculifolia *	PEE	*C. albicans *(ATCC 90028), 29* C. albicans *isolates from patients with vulvovaginal candidiasis	Inhibiting biofilm formation by *C. albicans* from vulvovaginal candidiasis	[149]
	Red propolis/*D. ecastaphyllum *	*n*-Hexane extract	5* C. parapsilosis *(RL01, RL07, RL11, RL13, and RL27), 5 *C. glabrata* (RL03, RL09, RL12, RL34, and RL37), *C. tropicalis* 72A, and *C. krusei *(ATCC 6258)	Active against fluconazole resistant *Candida *spp.	[148]

France	European propolis/*Populus nigra *	PEE, PWE, methanolic extract, and dichloromethane extract	*C. albicans* (ATCC 66396)*, C. glabrata *(LMA 90–1085), and* A. fumigates *(CBS 11326)	Antifungal activity against *C. albicans* and *C. glabrata* but only having a weak activity towards *A. fumigates *	[133]

Brazil	Green propolis/*B. dracunculifolia *	PEE	*P. brasiliensis*, peritoneal macrophages obtained from male BALB/c mice	Increase of fungicidal activity of macrophages against *P. brasiliensis *	[142]
Bulgaria	Mediterranean propolis/*Populus *spp.				

Czech Republic	European propolis/*Populus nigra *	Dimethyl sulfoxide extract	*C. albicans*, *A. fumigatus*, *M. gypseum*, and *M. canis *	Affecting the growth of the tested bacteria in different ways by different concentrations	[201]

Portugal (Bragança and Leiria)	European propolis/*Populus nigra *	PEE	*C. albicans, T. rubrum, *and *A. fumigatus *	Plant extracts not exhibiting relevant antifungal activity, but in general both propolis samples affecting the fungal growth	[140]

Poland	European propolis/*Populus nigra* and some species of *Betula alba, Alnus glutinosa, Aesculus hippocastanum, *and *Fagus sylvatica *	PEE	20 isolated *C. albicans, *14 isolated* C. glabrata, *and 10* C. krusei *	Inhibition of fungal growth	[147]

Iran	European propolis/*Poplar *spp*., Ferula ovina *	PEE	*C. albicans, C. tropicalis, C. kefyr, C. krusei, M. globosa, M. slooffiae, *and* M. pachydermatis*, all obtained from patients with clinical features of onychomycosis	Decrease of *Candida *and *Malassezia* strains growth, isolated from onychomycosis, even in the fluconazole-resistant strains	[205]

Brazil	Green propolis/*B. dracunculifolia *	PEE and propolis microparticles	89 yeast strains from vaginal exudates of the vulvovaginal candidiasis patients: 58 *C. albicans *and 17 *C. glabrata*, 1 *C. tropicalis*, 8 *C. guilliermondii*, and 5 *C. parapsilosis *	Inhibition of all yeasts growth by ethanol extract and propolis microparticles, with small variation, independent of the species of yeast	[144]
Argentina	Tropical region propolis*/Salix humboldtiana, Pinus *spp*., Eucalyptus *spp*.,* and* Populus *spp.	PEE	Xylophagous (*G. applanatum, L. elegans, P. sanguineus,* and* S. commune*) and phytopathogenic (*A. niger,Fusarium *sp*., Macrophomina *sp.,* P. notatum, *and *Rhodotorula *spp.)	Inhibition of fungal growth	[146]
Spain (Basque Country)	European propolis/*Populus nigra *	PEE and propylene glycol extracts	*C. albicans* (CECT 1394), *S. cerevisiae* (CECT 1383)	Inhibition of fungal growth	[145]

**Table 8:** Antitumoral activity of propolis and its chemical constituents.

Origin	Propolis type/plant source	Type of extract/isolated compound(s)	Species/cells	Effect	References
Thailand	Propolis from stingless bee *Trigona laeviceps *	Hexane extract	Colon (SW620), breast (BT474), hepatic (Hep-G2), lung (Chago), and stomach (Kato-III) cells Normal cell lines: liver (CH-liver) and fibroblast (HS-27)	High antiproliferative activity against the five cancer cell lines and low cytotoxic activity on the normal cell lines	[5]

Poland	European propolis/*Populus nigra* and some species of *Betula alba, Alnus glutinosa, Aesculus hippocastanum,* and* Fagus sylvatica *	PEE	Human malignant melanoma cell line Me45; colorectal cancer cell line HCT 116	Inhibition of cell growth and reduction of cell size of the tested cancer cells	[154]

Synthesized	Characteristic of propolis from the Pacific region, Thailand, Africa, Australia, and Brazil	Prenylated flavanones	Prostate cancer cell lines PC-3 and DU-145 Human hepatoma cell line Hep-3B	Induction of a more potent cytotoxicity against the PC-3 cell line than 5-flurouracil; induction of apoptosis	[45]
	Characteristic of European propolis	CAPE	C6 cell line established from a glioma generated by intravenous exposure of male Wistar rats to N-nitrosomethylurea BALB/c-nu mice	Inhibition of C6 glioma cells growth; increase in the percentage of cells in the G0/G1 phase, and decrease in the protein level of hyperphosphorylated pRb; increase in cyclin dependent kinase inhibitors p21, p27, and p16; decrease in tumor growth in xenografts, reduction of the number of mitotic cells and proliferating cell nuclear antigen- (PCNA-) positive cells in C6 glioma	[206]
			HL-60 cell line	Induction of apoptosis by activation of caspase-3, downregulation of Bcl-2, and upregulation of Bax	[207]

Brazil	Characteristic of Brazilian propolis	Drupanin, baccharin ((E)-3-prenyl-4-(2,3-dihydrocinnamoyloxy) cinnamic acid) and artepillin C (isolated from PEEs of propolis)	Human leukemia cell line HL-60, colon cancer cell line SW480	Inhibition of cells growth; promotion of morphological changes and nucleosomal DNA fragmentation (artepillin C > baccharin > drupanin)	[208]
	Red propolis/*D. ecastaphyllum *	Methanolic extract	Human pancreatic cancer cells (PANC-1)	Killing 100% of PANC-1 cells in the nutrient-deprived condition	[157]
	Green propolis/*B. dracunculifolia *	PEE	DU145 and PC-3 cell lines, telomerase-immortalized primary human prostate cancer-derived cell (RC-58T/h/SA#4), and primary human prostate epithelial cells (PrEC)	Inhibition of human prostate cancer cells proliferation by regulating the protein expression of cyclin D1, B1 and cyclin dependent kinase (CDK), p21	[158]
	Green propolis/*B. dracunculifolia *	Baccharin, beturetol, kaempferide, isosakuranetin, and drupanin (isolated from PEE)	Human embryonic kidney 293 (HEK293) cell, HCT116 cell line	Inhibition of HIF-1*α*, glucose transporter 1, hexokinase 2, and vascular endothelial growth factor A (VEGF-A) expression; exhibiting anti-angiogenic effects in the chick chorioallantoic membrane	[209]
	Green propolis/*B. dracunculifolia *	PEE	HUVECs cells	Induction of apoptosis in tube-forming endothelial cells through the inactivation of the survival signal ERK1/2 and by the activation of caspase-3	[175]
	Green propolis/*B. dracunculifolia *	PWE	Female Wistar rats	Inhibition of angiogenesis in N-butyl-(-4-hydroxybutyl) nitrosamine- (BBN-) induced rat bladder cancer	[210]
	Red propolis/*D. ecastaphyllum *	PEE	Human immortalized endothelial-like cell line EA.hy926, renal cell carcinoma cell line RCC4, and mouse embryonic stem cell line CGR	Reduction of migration and sprouting of endothelial cells and attenuation of new blood vessels formation; decrease in the differentiation of embryonic stem cells into CD31 positive cells; decrease in HIF1-*α* protein accumulation which attenuates VEGF gene expression, increases the von Hippel-Lindau- (pVHL-) dependent proteasomal degradation of HIF1-*α*, and downregulates Cdc42 protein expression	[211]

Brazil	Green propolis/*B. dracunculifolia *	PEE	Human umbilical vein endothelial cells (HUVECs), NF1-deficient MPNST (S-462), and NF2-deficient schwannoma (HEI-193) cell lines Female nu/nu mice	Blocking PAK1 signaling selectively, without affecting AKT; suppressing almost completely the growth of human neurofibromatosis tumor xenografts in mice	[156]
Synthesized	Characteristic of Brazilian propolis	Artepillin C			

Brazil	Green propolis/*B. dracunculifolia *	PEE	HUVECs Female ICR mice	Reduction of the number of newly formed vessels *in vivo* Suppression of HUVECs proliferation and inhibition of tube formation	[174]
Purchased: Wako Pure Chemicals Industries (Osaka, Japan)	Characteristic of Brazilian propolis	Artepillin C			

Brazil	Green propolis/*B. dracunculifolia *	PEE	LNCaP cell line	Sensitizing TRAIL-resistant LNCaP cells to TRAIL-induced apoptosis Induction of a significant disruption of ΔΨm Enhancing the expression of TRAIL-R2 and decreasing the activity of NF-*κ*B Artepillin C, quercetin, kaempferol, and *p*-coumaric acid strongly cooperating with TRAIL to induce apoptosis	[167]
Purchased: Alexis Biochemicals (San Diego, CA, USA)	Characteristic of Brazilian propolis	Quercetin, kaempferol, and *p*-coumaric acid			
Purchased: Wako Pure Chemicals (Osaka, Japan)		Artepillin C			
Brazil	Red propolis/*D. ecastaphyllum *	PEE	MCF-7 cell line	Reducing cell viability through induction of mitochondrial dysfunction, caspase-3 activity, and DNA fragmentation and increase in expression of CCAAT/enhancer-binding protein homologous protein (CHOP)	[212]
Purchased: Api Co. Ltd., Gifu, Japan	Characteristic of European propolis	CAPE			

Purchased: Sigma Aldrich Co.	Characteristic of European propolis	CAPE	LNCaP 104-S, DU-145, and PC-3 cell lines Male BALB/c mice	Suppressing the growth of LNCaP, DU-145, and PC-3 and inhibiting the tumor growth of LNCaP xenografts, possible inhibition of p70S6K and some Akt signaling networks	[161]
			PC-3 cell line	Suppressing proliferation, colony formation, and cell cycle progression, decrease in protein expression of cyclin D1, cyclin E, SKP2, c-Myc, Akt1, Akt2, Akt3, total Akt, mTOR, and Bcl-2, Rb, as well as phosphorylation of Rb, ERK1/2, Akt, mTOR, GSK3a, GSK3b, and PDK1, and increase in KLF6 and p21^Cip1^ protein expression	[213]
			Breast cell lines MDA-MB-231, MCF-7, MCF-10A, and MCF-12A Bovine capillary endothelial (BCE) cells Female (Ncr-nu-nu) mice	Inhibition of *in vitro* and *in vivo* MCF-7 and MDA-MB-231 tumor growth without much effect on normal mammary cells; induction of cell cycle arrest and apoptosis by downregulation of Bcl-2 proteins; reduction of NF-*κ*B and mdr-1 gene expression and suppression of VEGF production by MDA-231 cells and formation of capillary-like tubes by endothelial cells	[162]
			Breast cancer cell lines MDA-MB-231, MCF-7, and SKBR3	Promotion of an accumulation of acetylated histone proteins in MCF-7 (ER+) and MDA-MB-231 (ER−/PR−/Her2−); decrease of ER and PR in MCF-7 cells and upregulation of ER and decrease of EGFR in MDA-MB-231 cells; reduction of Her2 protein in SKBR3 (Her2+) cells	[163]
			Human oral squamous cell carcinoma TW2.6	Suppression of cell proliferation and colony formation; decrease of G1 phase cell population, increase of G2/M phase cell population; induction of apoptosis; decrease of Akt, Akt1, Akt2, Akt3, phospho-Akt Ser473, phospho-Akt Thr 308, GSK3*β*, FOXO1, FOXO3a, phospho-FOXO1 Thr24, phospho-FoxO3a Thr32, NF-*κ*B, phospho-NF-*κ*B Ser536, Rb, phospho-Rb Ser807/811, Skp2, and cyclin D1; and increase of cell cycle inhibitor p27^Kip^	[214]
			MDA-MB-231 cell line	Inhibition of cancer stem cells self-renewal, progenitor formation, and clonal growth and decrease of CD44 levels	[164]
			Human hepatocellular carcinoma cell line SK-Hep1	Suppression of the adhesion and invasion potential of the cells by inhibiting completely the expression of MMP-2 and metalloproteinase-9 (MMP-9) and the NF-*κ*B	[172]
			Human fibrosarcoma cell line HT1080	Decrease of MMP and tissue inhibitor metalloproteinase-2 (TIMP-2) mRNA levels; downregulation of MMP-2 and MMP-9 expression; inhibition of MMP-2 activity; decrease of invasion, motility, cell migration, and colony formation	[215]

Turkey	Mediterranean propolis*/Populus *spp*., Eucalyptus *spp., and* Castanea sativa *	PEE	MCF-7 cell line	Increase of apoptosis through the caspase pathway	[216]

Iraq	European propolis/*Populus nigra *	PWE	HL-60 cell line, colon cancer cell HCT-116 Female athymic Fox N1-nu/nu mice	Inhibition of HL-60 cells proliferation and induction of apoptosis by downregulating Bcl-2 protein and upregulating Bax; inhibition of HCT-116 cells colony formation potential and promotion of necrotic changes; decrease of mitotic cells and increase of p53 and Ki-67 expression in HCT-116 tumor-bearing mice	[13]

Iran	European propolis/*Poplar *spp*., Ferula ovina *	PEE	Male Wistar rats	Decrease of tumour incidence, number of lesions, structural abnormalities, and beta-catenin and induction of proapoptotic Bax expression and reduction of antiapoptotic Bcl-2 expression	[165]

Provided by Wako Pure Chemicals (Osaka, Japan)	Characteristic of Brazilian propolis	Artepillin C	LNCaP cell line	Induction of caspase-8 and caspase-3 activation and disruption of mitochondrial membrane potential by a cotreatment with TRAIL and artepillin C	[169]

Purchased	Characteristic of European propolis	CAPE	MCF-7 cell line	Induction of apoptosis via Fas signal; induction of Bax protein and activation of caspases and MAPK family proteins p38 and JNK	[217]

Purchased: Calbiochem (San Diego, CA, USA)	Characteristic of European propolis	CAPE	Lymphoblastoid cell line PL104	Induction of apoptosis through phosphatidylserine (PS) exposure and nuclear fragmentation Increase of sub-G1 DNA content; downregulation of surviving and Bcl-2 expression and increase of BAX proteins levels; induction of mitochondrial membrane potential (Δ*ψ*m) collapse; induction of cytochrome c release from mitochondria and induction of caspases 3, 7, and 9 activation	[171]

China;	European propolis/*Populus nigra *	PEE	HUVECs	Inhibition of VEGF expression	[179]
Purchased: Api Co. Ltd. (Gifu, Japan)	Characteristic of European propolis	CAPE			

Korea;	European propolis/*Populus nigra *	PEE	Fertilized chicken eggs Calf pulmonary arterial endothelial (CPAE) cells	Inhibition of angiogenesis in chick embryo chorioallantoic membrane and inhibition of CPAE cells proliferation	[176]
Synthesized	Characteristic of European propolis	CAPE			

Portugal: (Serra de Bornes and Fundão);	European propolis/*Populus nigra *	Methanolic extract	Normal and cancerous renal cells derived from human renal cell carcinoma patients, human renal carcinoma cell line A-498	Inhibition tumor cells growth exhibiting selective toxicity against malignant cells compared to normal cells	[64]
(Anga do Heroísmo, Azores)		PEE and hexane, chloroform, and residual ethanol extract fractions obtained from the PEE	Human colorectal adenocarcinoma cell line HCT-15	All the samples exhibiting cytotoxic effect against tumor cells; chloroform fraction decreasing cell viability, promoting cell death, and disturbing tumor cell glycolytic metabolism	[218]

## Abbreviations

CAPE: Caffeic acid phenethyl ester
PEE: Propolis ethanol extract
PWE: Propolis water extract
RNS: Reactive nitrogen species
ROS: Reactive oxygen species
VEGF: Vascular endothelial growth factor.
